# Venom-based peptide therapy: insights into anti-cancer mechanism

**DOI:** 10.18632/oncotarget.21740

**Published:** 2017-10-11

**Authors:** Rui Ma, Ravikiran Mahadevappa, Hang Fai Kwok

**Affiliations:** ^1^ Faculty of Health Sciences, University of Macau, Avenida de Universidade, Taipa, Macau SAR

**Keywords:** anticancer mechanism, venom, metastasis, targeted therapy, signaling pathway

## Abstract

The 5-year relative survival rate of all types of cancer has increased significantly over the past three decades partly due to the targeted therapy. However, still there are many targeted therapy drugs could play a role only in a portion of cancer patients with specific molecular alternation. It is necessary to continue to develop new biological agents which could be used alone and/or in combination with current FDA approved drugs to treat complex cancer diseases. Venom-based drugs have been used for hundreds of years in human history. Nevertheless, the venom-origin of the anti-cancer drug do rarely appear in the pharmaceutical market; and this is due to the fact that the mechanism of action for a large number of the venom drug such as venom-based peptide is not clearly understood. In this review, we focus on discussing some identified venom-based peptides and their anti-cancer mechanisms including the blockade of cancer cell proliferation, invasion, angiogenesis, and metastasis (hallmarks of cancer) to fulfill the gap which is hindering their use in cancer therapy. Furthermore, it also highlights the importance of immunotherapy based on venom peptide. Overall, this review provides readers for further understanding the mechanism of venom peptide and elaborates on the need to explore peptide-based therapeutic strategies.

## INTRODUCTION

According to the Global Cancer Statistics published in 2015, there are approximately 32.6 million cancer patients around the world in 2012 [1]. It is no doubt that cancer is one of the primary causes of death in the world [2]. Over the last three decades, the 5-year relative survival rate for all type of cancers has increased significantly, and this is partially due to the successful development of targeted therapy [3]. However, still many of the current targeted therapeutic drugs could only work in a portion of patients who carried specific molecular alternations. There is a necessity to continuously develop some new biologics which could work alone and/or in a combination with the current FDA approved drugs to fight against the complex cancer disease.

One of the exciting developments in the field of anti-cancer research is the isolation of cancer specific and anti-proliferative drugs from animal venoms. High specificity and selectivity towards proteins and protein sub-types have made venom an invaluable source of future drugs in fighting cancer. The venom-based drug is not a new idea, and it has been used in human history for more than hundreds of years, for example, use of snake venom against arthritis and dried toad skin secretions against pain are well described in Chinese traditional medicine and Indian Ayurvedic medicine for centuries [4]. In the past three decades, natural products such as secretion from plants and venom/secretion from animals play one of the major sources of novel drug design and development [5]. Currently, many venom peptide drugs are available in the market for treatment of diseases such as cardiovascular disease, diabetes, hypertension, multiple sclerosis, and pain [6]. Venom is a concoction of toxins which are the nature’s most efficient cytotoxic agent. “Venom” is defined as “A secretion produced by specialized cells in one animal, delivered to a target animal through the infliction of a wound and that disrupts endo-physiological or biochemical processes in the receiving animal to facilitate feeding, defense or competition by/of the producing animal” [7]. Although the words “venom” and “toxin” are used in parallel in some context, venom is injected or delivered through animal/organism bites whereas toxins are ingested. Venomous animals are widely present in nature from Arthropoda phylum (Arachnids: Spiders, Scorpions) to Cnidaria (Anthozoa: Sea Anemones) [4]. The venom of a single species may contain hundreds to several thousands of active peptides evolved by natural selection [8, 9]. Tremendous variation and molecular diversity of venom have opened up whole new avenues for future pharmacology.

Characteristic pharmacological properties of peptides such as specificity, selectivity, stability, smaller size (around 10–80 residues) make peptides an ideal candidate and a possible spear-head for fighting cancer in future. Venom peptides are disulfide-rich molecules which exhibit high-affinity target binding compared to synthetic peptides. However, complete utilization of venom-based peptide in medicine is still staggering due to the fact that the action mechanism of a large number of the venom-based peptide is not clearly understood. In this direction, our research group works extensively on understanding venom-based peptide interactions and molecular mechanism in cancer. Recent reviews have discussed the advantages and development possibilities of drugs based on venom from different venomous species [10–12]. In this review, we focus on currently identified venom-based peptides and its anti-cancer mechanisms to fulfill the gap which is hindering its use in cancer therapy and also examines the potential use of venom-based peptides as an effective anti-cancerous drug. Furthermore, it also emphasizes the importance of immunotherapy based on venom-peptides. Taken together, this review provides the reader further understanding on action mechanism of venom peptides along with elaborating the need in exploring peptide based treatments.

## VENOM-BASED PEPTIDE DRUGS

**Figure 1:**
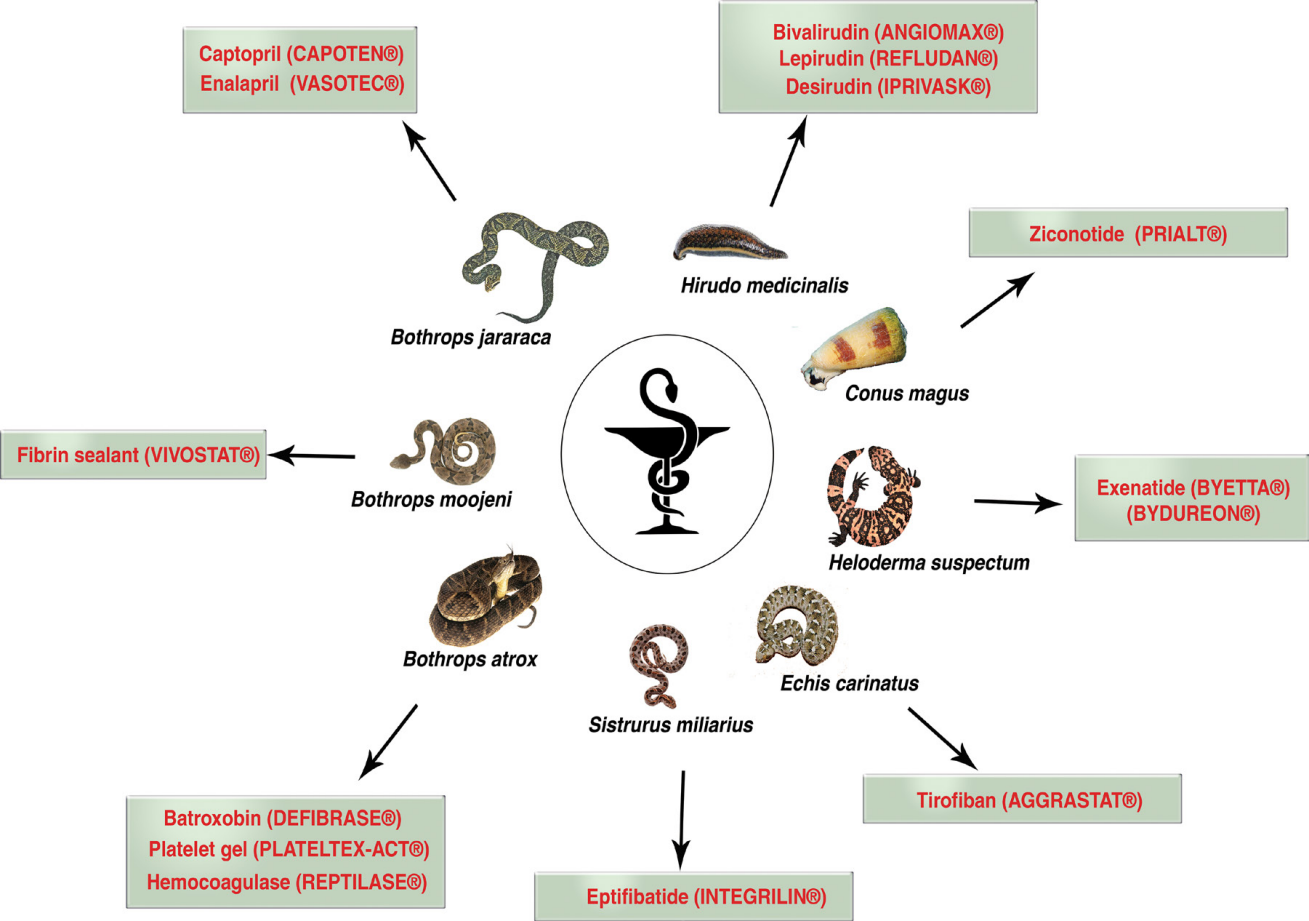
Current venom-based drugs in the market used for different forms of human disease

Owing to the fact of its toxic nature, today, only a few handful venom-based drugs are approved for its use and currently in the market (Figure 1). A breakthrough in use of venom-peptide came to light after the development of captopril, an anti-hypertensive drug [13]. Captopril, an analogue of dipeptide Ala-Pro from snake *Bothrops jararaca* effectively bind to the active site of Angiotensin-converting enzyme (ACE). ACE a key enzyme of renin-angiotensin system that converts angiotensin I to an active vasoconstrictor angiotensin II which regulates the volume of fluids in blood. Captopril an active ACE inhibitor is used in the treatment of hypertension. Following captopril footsteps, a tri-peptide Phe-Ala-Pro analogue enalapril was also developed [13]. Captopril was approved for its use in 1981, and since then many venom-peptide or venom-peptide analogues have been tested for various disease with few success (Table 1) [6]. Table 1 depicts various venom-based drug brands in the market today and its application against various disease and its application against various disease conditions along with mechanism of actions.

**Table 1:** Mechanism of action of some of the venom based drugs currently available in the market[6]

Generic name (BRAND NAME)	Mechanism of action	Indication (Diseases)
Captopril (CAPOTEN^®^)	Angiotensin-converting enzyme inhibitor	Hypertension, Cardiac failure
Enalapril (VASOTEC^®^)		
Exenatide (BYETTA^®^) (BYDUREON^®^)	Glucagon-like peptide-1 receptor agonist	Type 2 diabetes mellitus
Ziconotide (PRIALT^®^)	Ca_v_2.2 channel antagonist	Management of severe chronic pain
Bivalirudin (ANGIOMAX^®^)	Reversible direct thrombin inhibitor	Anticoagulant in percutaneous coronary intervention
Lepirudin (REFLUDAN^®^)	Binds irreversibly to thrombin	Anticoagulation in heparin-associated thrombocytopenia; Thromboembolic disease
Desirudin (IPRIVASK^®^)		
Tirofiban (AGGRASTAT^®^)	Prevents binding of fibrinogen, von Willebrand factor, and other adhesive ligands to GPIIb/IIIa	Acute coronary syndrome; Percutaneous coronary intervention
Eptifibatide (INTEGRILIN^®^)		
Batroxobin (DEFIBRASE^®^)	Cleaves Aα-chain of fibrinogen	Acute cerebral infarction; unspecific angina pectoris; Sudden deafness; Gelification of blood for topical applications
Platelet gel (PLATELTEX-ACT^®^)		
Hemocoagulase (REPTILASE^®^)	Fibrinogenase	Prophylaxis and treatment of hemorrhage in surgery
Fibrin sealant (VIVOSTAT^®^)	Cleaves Aα-chain of fibrinogen; factor X and/or prothrombin activation	Autologous fibrin sealant in surgery

^*^The source is from [6].

Many technical advances during last decade have exemplified the importance of venom-peptide in drug discovery. Venom is a complex mixture of proteins, peptides, enzymes and non-protein inclusions [14]. Today, using advanced proteomics and genomics approach it is possible to isolate and characterize the potential anti-cancer peptides from venom pool. Further, structural analysis of isolated peptides and its interactions with protein or target molecule has revealed specific amino-acid domains that exhibit anti-proliferative effects, for example – importance of RGD domains in peptide including disintegrins family. RGD sequence presents in most of the disintegrins isolated from snake species and provides a structural scaffold for interactions with transmembrane receptor integrins (importance of disintegrins in anti-cancer therapy is discussed later in this review). Structural modifications of such domain lead to increase stability and elimination of liable peptide bonds that may comprise a peptide to enzymatic degradation. Venom-based peptides being small and easily modified, the prospect of using them or their analogues in cancer therapy is promising.

## ANTI-CANCER MECHANISM OF VENOM PEPTIDES

Compared to normal cells, cancer cells have the ability to circumvent the cell cycle checkpoint, responsible for maintaining intracellular balance *in vivo* [15]. Although the multistep process of cancer development is divided into three physiological stages, i.e., initiation, promotion, and progression of cancer, the distinction between the three stages in the dimension of time is artifactual. In a leading edge review on cancer by Hanahan and Weinberg, authors discuss six important hallmarks of cancer that provides a logical framework for understanding the chronic process of cancer [16]. Hallmarks of cancer include sustaining proliferative signaling, evading growth suppressors, activating invasion and metastasis, enabling replication immortality, inducing angiogenesis, and resisting cell death. Besides, there is the introduction of two emerging hallmarks including deregulating cellular energetics and avoiding immune destruction [16]. When normal cells acquire the sustaining proliferative signaling, they will enable to get other hallmarks to become tumorigenic. So an ideal anti-cancer drug would be able to inhibit and/or block any one or some of the hallmarks.

The anti-cancer mechanism of peptides is no exception to inhibit and/or block these hallmarks (Table 2). Table 2 lists some venomous peptides and indirectly derived drugs, which shows their molecular targets and distinct anti-cancer mechanisms. Recent studies have revealed many novel modes of anti-cancer mechanism beyond our previous understanding of venom peptides in membrane pore formation. Recent studies have unveiled the interaction of venom peptides with membrane receptor molecules and non-receptor components, extracellular matrix, etc. And then these interactions can affect several cell signaling pathways, and cell organelles such as endoplasmic reticulum or mitochondria which were damaging the host cell to initiate the death signals.

**Table 2:** The anticancer mechanisms of some venomous peptides and indirectly derived drugs

Target	The major mechanisms of action	Molecular target		Drug	Drug class	Indications	Clinical phase	Reference
Ion channels	The proliferation and invasion of cancer cells	Chloride (Cl^-^) channels: CLC 3		^131^I-TM601 (^131^I-CTX)	peptide(36aa)	Gliomas	Phase III	[76, 77]
				BLZ-100 (ICG-CTX)	peptide(36aa)	Gliomas tumor marker for surgery	Phase I	[67]
		Sodium (Na^+^) channels		AGAP	peptide(66aa)	Colon cancer cells, Malignant glioma cells	Preclinical studies	[88, 89]
		Potassium (K^+^) channels: K_V_11.1(hERG)		Ergtoxin	peptide (42- 62aa)	Ovarian cancer cells	Preclinical studies	[93]
		Transient receptor potential (TRP) channels: TRPV6		SOR-C13	peptide(13aa)	Solid tumors with overexpressing the TRPV6 ion channel	Phase I	[99]
Integrins	The invasion, migration, angiogenesis, and metastasis of cancer cells	α_v_β_3_, α_v_β_5_		Cilengitide	Peptidomimetic (5aa)	1 Glioblastoma with methylated MGMT promoter 2 Glioblastoma with unmethylated MGMT promoter 3 NSCLC	1 Phase III 2 Phase II 3 Phase II	[104]
		α_5_β_1_		ATN-161	Peptidomimetic	Malignant Glioma	Phase II	[133]
		Five integrin receptors (α_v_β_1_, α_v_β_3_, α_v_β_5_, α_v_β_6_, α_5_β_1_)		GLPG0187	Peptidomimetic	Bone metastasis in metastatic breast cancer	Phase I	[134]
		α_v_β_3_, α_v_β_5_, α_5_β_1_		Vicrostatin	peptide(69aa)	Ovarian cancer, Gliomas	Preclinical studies	[130–132]
G protein-coupled receptor	The metastasis of cancer cells	Gastrin-releasing peptide receptor		BAY86-7548	peptide(14aa)	Prostate cancer imaging	Phase II/III	[137]
Membrane molecules	The disruption of cancer cell membrane		Sialic acid-rich glycoproteins,PS and PC,heparan sulfate	1 MP1 2 Melittin 3 Mastoparan	1 peptide(14aa) 2 peptide(26aa) 3 peptide(14aa)	1 Human leukemic Jurkat cells 2 Human renal cancer, lung cancer, liver cancer, etc. 3 Pancreatic cancer cells	Preclinical studies	[26–28][30–33][34]
			Phospholipids	Hemilipin	heterodimer	HUVECs and HPAECs	Preclinical studies	[40]

^*^BLZ-100: Chlorotoxin-indocyanine green Imaging Agent; CTX: chlorotoxin; ICG: dye indocyanine green; AGAP: Analgesic-Antitumor Peptide; hERG is a gene (*KCNH2*) that codes for potassium channels K_v_11.1; Ergtoxin is a family of toxins from the venom of the Mexican scorpion; MGMT: O^6^-alkylguanine DNA alkyltransferase; TRPV (“V” for vanilloid); PS: phosphatidylserine; PE: phosphatidylethanolamine; NSCLC: Non-Small Cell Lung Cancer; Hemilipin is a novel sPLA2; HUVECs: Human Umbilical Vein Endothelial Cells; HPAECs: Human Pulmonary Artery Endothelial Cells.

### Interactions with cancer cell membrane

#### Disruption of plasma membrane

At cell membrane level, cancer cells differ from normal cells by two factors, i.e., an increased net negative charge and a higher number of microvilli which increases the surface area of cancer cells. In normal mammalian cells, the anionic phosphatidylserine (PS) and phosphatidylethanolamine (PE) are found in the inner membrane, and zwitterionic phospholipids are in outer membrane [17–19]. However, upon transformation of the normal cell to a cancer cell, cell membrane will lose the asymmetric transmembrane distribution of phospholipids where a percentage of PS and PE will be transported in the outer monolayer thereby increasing the net negative charge. Increased negative charge in cancer cells is also due to an elevated expression of anionic molecules such as O-glycosylated mucins (high molecular weight O-glycoside with negatively charged saccharides), gangliosides, and heparin sulfides on the outer layer of membrane. Some venom peptides are a part of antimicrobial peptides (AMPs, also called host defense peptides). Usually, these peptides are relatively smaller (12–50 amino acids), a large proportion (generally >30%) of hydrophobic residues and have a net positive charge from +2 to +9 due to the presence of multiple arginine, lysine, and histidine [20]. These short peptides can form four types of secondary structures: α-helical, β-stranded, β-loop, and extended [21]. The most venom antimicrobial peptides belong to the α-helical type, such as melittin and mastoparan, etc. Some peptides are unstructured in the buffer and fold into their final secondary configuration when binding to the cell membrane. Usually, circular dichroism and solid-state NMR spectroscopy are used to measure the orientation and secondary structure of an antimicrobial peptide bound to a lipid bilayer [22]. These different characters between cancer cells and antimicrobial peptide promote electrostatic interactions thereby increasing the cancer-selective toxicity. Coupled with the hydrophobic interaction of hydrophobic amino acids, amphiphilic antimicrobial peptides are more likely to be inserted into the membrane phospholipid bilayer [23]. Once bound to the cell membrane, peptides execute a cytotoxic action by disruption of cell membrane either by pore formation (the barrel-stave model or the carpet model or the toroidal model, etc.) [24] or membrane disruption or disaggregation of membrane lipids by micelles formation. Meanwhile, the combination of cationic peptides increases the transmembrane potential, which is more favorable for membrane permeabilization. Fluorescent dyes are used to be a common method to measure the ability of antimicrobial peptides to form membrane pore.

#### Polybia-MP1

Polybia-MP1 was isolated from the venom of the Brazilian wasp *Polybia Paulista* [25]. It is a 1.6 kDa peptide (primary structure: IDWKKLLDAAKQIL-NH2) with an amidated C-terminal residues form [26]. Smaller size, cationic nature (a net positive charge of +2) and more than 30% of hydrophobic amino acids contribute to the formation of amphipathic and helical conformations, which have the ability to interact electrostatically with the anionic components of the membranes to form a pore-like structure [26]. Polybia-MP1 selectively inhibits proliferating bladder and prostate cancer cells, multidrug-resistant leukemic cells, and leukemic T-lymphocytes without being hemolytic and cytotoxic [26–28]. In parallel replacement of Leu7, Asp8 or Ala9 disrupts alpha helix conformation indicating the importance of alpha-helix conformation for its anti-tumor activity [29]. Toxic nature of polybia-MP1 against human leukemic Jurkat cells was analyzed using bilayer membrane models [28]. Polybia-MP1 induced pore-forming activity on membranes with bilayers formed by a mixture of phosphatidylcholine and phosphatidylserine (70:30) with a high content of anionic lipids [28]. The pore-forming activity of MP1 was reduced with the addition of less charged cholesterol molecules into the membrane. These observations pointed to the fact that induced cytotoxicity of polybia-MP1 is due to membrane pore formation and not genotoxicity [28].

### Disruption of plasma membrane and mitochondrial membrane

Increased surface area of cancer cells (due to a greater number of microvilli) also enhances the amount of internalization of membrane-bound peptides. Melittin induces to membrane pore formation by the toroidal model [24]. Internalized peptides can further interact with mitochondrial membrane causing a transition pore across the mitochondrial inner membrane. Such pore condition makes the inner membrane permeable to cytosolic ions and solutes inducing swelling and rupture of mitochondria. The release of cytochrome c from mitochondria causes a cascade of reactions thereby activating apoptotic pathway within the cell.

#### Melittin

**Figure 2:**
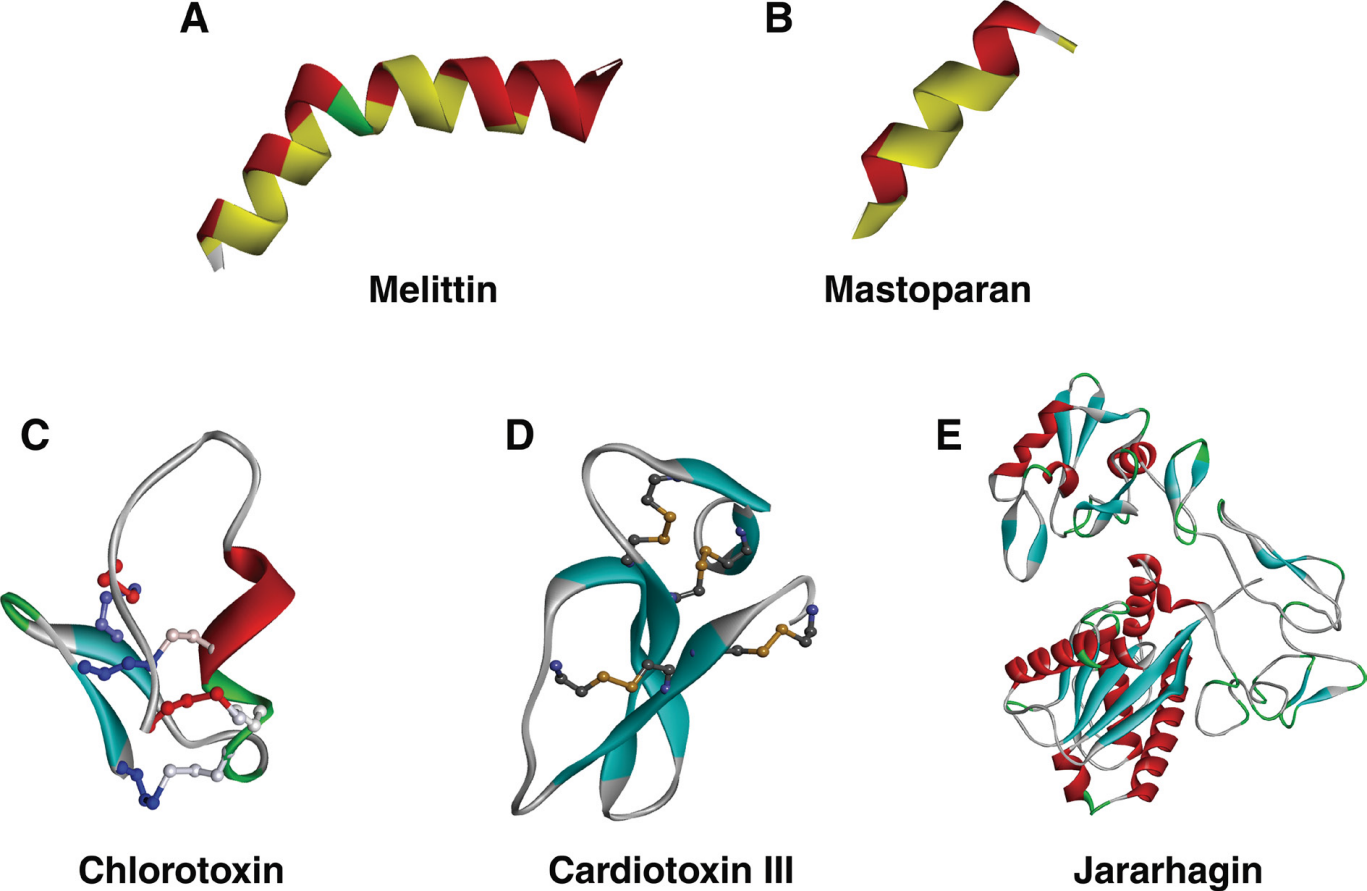
3D structure of venom peptides (**A** and **B**) Amphipathic peptides melittin (PDB ID:2MLT) and mastoparan (PDB ID: 2CZP) exhibiting the increase of hydrophobicity in the alpha helix (yellow region), respectively. (**C** and **D**) Another key feature of venom peptides, disulfide bridges show in ion channel blocker chlorotoxin (PDB ID: 1CHL) and mitochondrial membrane binding peptide cardiotoxin III (PDB ID: 2CRT), respectively. (**E**) Jararhagin, a metalloprotease with multiple alpha helices and beta sheets and its 3D structure is modeled from SWISS-MODEL. Figures were constructed by Discovery Studio version 2016.

Melittin, a major peptide in the venom of bee *Apis mellifera,* is known to exhibit anti-tumor effect predominantly through mitochondria-mediated apoptosis. Melittin has shown antitumor roles against human renal cancer, lung cancer, liver cancer, prostate cancer, bladder cancer, mammary cancer, and leukemia. Melittin, a 2.8 kDa peptide consists of 26 amino acids with five basic amino acids (three Lys and two Arg). Melittin with a net charge of +6 exhibits an alpha helix conformation (Figure 2A) [30]. Its amphipathic structure can permeate cell membranes to form the pores. Studies using human gastric cancer cells SGC-7901 showed that melittin increased mitochondrial membrane potential, thereby releasing pro-apoptotic factors such as Cyt C, apoptosis inducing factors, EndoG, and Smac/Diablo [31]. This phenomenon was also reflected when SGC-7901 was observed under transition electron microscope which showed decreased microvilli, chromatin condensation, enlarged perinuclear space, cristae and membrane dissolution and formation of apoptotic bodies which are typical for mitochondrial-induced apoptosis [31]. Furthermore, the studies also showed an increase in reactive oxygen species leading to oxidative damage within the cell. In human peripheral blood lymphocytes (HPBLs), melittin exhibited genotoxic effects by modulating expression of DNA damage response such as TP53, CDKN1A, GADD45α, and MDM2; oxidative stress such as CAT, SOD1, GPX1, GSR, and GCLC; and also apoptotic markers BAX, BCL-2, CAS-3, and CAS-7 [32]. Advanced research using bifunctional fusion protein of melittin and interleukin-2 (melittin-IL-2) showed an increased cytotoxicity in human liver (SMMC-7721 cancer cells), lung (A549 cancer cells), and ovarian (SKOV3 cancer cells) cancer xenograft models [33]. Although more studies are required to analyze its efficiency, melittin is proving to become a potent drug in future.

#### Mastoparan

Mastoparan is another membrane spanning cationic peptide (14 amino acids) which is also known to induce mitochondrial-mediated apoptosis (Figure 2B) [34]. Mastoparan obtained from wasp *Vespula lewisii* venom exhibits cytotoxic effect against mast cells, pancreatic cancer cells, platelets and also known to induce expression of phospholipase A2 and C [34]. Similar to melittin, mastoparan is known to disrupt mitochondrial membrane potential and generate reactive oxygen species, thereby executing mitochondrial-induced apoptosis pathway. Importantly, mastoparan reduced the growth of melanoma *in vivo* and increased mice survival [34].

### Interactions with membrane phospholipids

#### Phospholipase A2 - Hemilipin

**Figure 3:**
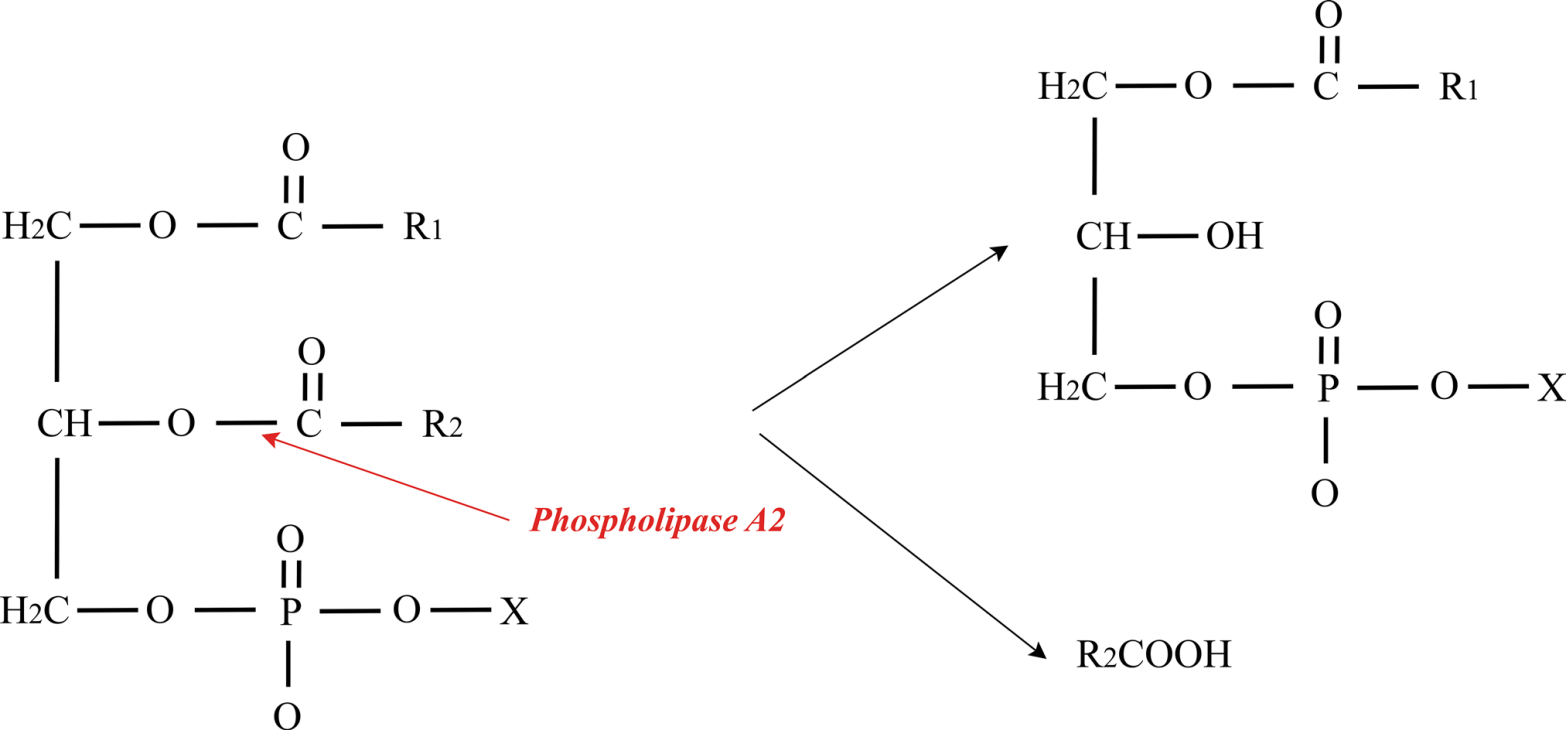
Schematic representation of PLA2 action on glycerophospholipids PLA2 enzymes catalyze the hydrolysis of the sn-2 ester bond in glycerophospholipids to produce free fatty acids and lysophospholipids.

Phospholipases A (2) (PLA2s) EC 3.1.1.4 are enzymes which can catalyze the hydrolysis of the sn-2 ester bond in phospholipids to produce free fatty acids and lysophospholipids (Figure 3). PLA2s release biologically active fatty acids and lysophospholipids from plasma membrane that is important autocrine and paracrine regulators during cancer progression [35]. PLA2s also exhibit a broad range of physiological and pathological effects [36]. Based on their molecular weight, primary structure, localization, and calcium requirement for enzymatic activity, PLA2s are classified into six types: secreted (sPLA2), cytosolic (cPLA2), calcium-independent (iPLA2), platelet activating factor acetylhydrolase (PAF-AH), lysosomal PLA2 (LPLA2), and adipose-PLA2 (AdPLA) [37]. The sPLA2 is the most common types of PLA2 discovered in the snake venom with the low molecular weight of 13–19 kDa which contains high disulfide bridge content [38]. The sPLA2 can be found in the venom of both vertebrates and invertebrates, and their active sites have a conserved histidine/aspartates dyad which depends on calcium for their catalytic activity [39].

Hemilipin is a novel sPLA2, extracted from *Hemiscorpius lepturus* scorpion venom. In normal human umbilical vein endothelial cells (HUVECs) and human pulmonary artery endothelial cells (HPAECs), hemilipin inhibited angiogenesis without exhibiting cytotoxic effects [40]. When HUVECs and HPAECs seeded onto matrigel with cytokines VEGF/FGF-2 *in-vitro* analysis, hemilipin firmly blocked VEGF/FGF-2 induced angiogenesis at 50nM concentration. Similarly, hemilipin also showed anti-angiogenic effects *in-vivo* chorioallantoic membrane (CAM) assay. However, the concentration was ten times higher than the level *in-vitro* analysis. In parallel, scratch wound assay performed using HUVECs and HPAECs also confirmed the ability of hemilipin to inhibit cell migration. It is hypothesized that hemilipin exhibits its anti-angiogenic effects by reducing the growth factors such as VEGF isoforms (VEGF-A, VEGF-C, VEGF-D) and its receptors such as VEGFR-1 and VEGFR-2.

### Interactions with membrane carbohydrates

#### Lectins

Lectin is a family of proteins that have the high binding specificity for carbohydrates. Lectins are extensively existing in nature and are found in animals, plants, bacteria, and viruses [41]. According to the primary structure of animal lectins, it can be divided into 10 families at least including C-type, S-type (galectins), I-type (siglecs and others), P-type (phosphomannosyl receptors), Pentraxins and fibrinogen-type, etc [42]. The activity of C-type lectins requires Ca^2+^ ions and the activity of S-type depends on the free sulfydryl [43], which get a more extensive study. One of the malignant cells’ remarkable features is the change of glycoconjugate patterns on the cell surface [44]. Different lectins have the ability to recognize carbohydrate domains of glycoproteins and/or glycolipids in the cell membrane [45], therefore lectins have developed as histochemical probes to describe alterations in cancer cell surface.

BJcul, a lectin purified from the snake *Bothrops jararacussu* venom, is disulfide-linker homodimer composed of 15 kDa subunits [46]. It has a carbohydrate binding specificity for β-galactose, belonging to the C-type lectin with calcium binding zone. The crystallographic structure of BJcuL has been resolved, which reveals a porous and flexible decameric conformation composed of five dimers [47]. BJcuL is able to interact with glycoligands targets on the surface of gastric carcinoma cells MKN45 and AGS, and decrease their viability and adhesion, at last result in cytotoxic effects [48]. To evaluate the apoptosis mechanisms of BJcuL, research has been carried out by studying the BJcuL interacting with HT29 human colon adenocarcinoma cells [49]. Result has demonstrated a dose-dependently cytotoxic effect was inhibited in the presence of D-galactose. The BJcuL has the ability to unregulate TRAIL (Tumour necrosis factor-related apoptosis-inducing ligand) expression, as a result of increasing in the expression of apoptosis-related proteins, such as FADD, caspase-8, and Bax [49]. A study has shown that the growth of eight cancer cell lines were inhibited by BJcuL lectin in a dose-dependent manner: IC50 of renal cells (Caki-1 and A-498) and pancreas (CFPAC-1) cancer cell lines were as low as 1–2 mM; and IC50 of melanoma (Wm115) and prostate (PC-3) cancer cells were 7.9 and 8.5 mM, respectively [50].

Lebecetin, a C-lectin protein purified from the venom of *Macrovipera lebetina* snake, is a disulfide-linked heterodimer of 15 and 16 kD subunits [51]. Lebecetin belongs to a “bifunctional lectin molecule” which can bind to non-carbohydrate molecules such as protein, lipid, and nucleic acid in addition to carbohydrates [42]. Lebecetin can bind the α_5_β_1_ and αv-containing integrins and displays anti-integrin activity including cell adhesion, migration, invasion, and proliferation [52]; and it also can inhibit the adhesion of IGR39 melanoma and HT29D4 adenocarcinoma cells [51]. Another lectin named lebectin, a C-type lectin of 30 kDa heterodimer from *Macrovipera lebetina* venom, has been reported to inhibit the α_5_β_1_ and α_v_-containing integrins [52]. Another study also showed that lebecin can inhibit the proliferation of MDA-MB231 human breast cancer cells [53]. Furthermore, lebecin also blocks the migration of MDA-MB231 cells in haptotaxis assays. Lectins vixapatin [54] and rhodocetin [55] were characterized as a selective α_2_β_1_ integrin antagonist.

### Interactions with membrane receptor molecules

Metastasis of cancer cells is the leading cause of increased mortality in cancer patients [56, 57]. As the tumor grows slowly (usually less than 2 mm^3^), the tumor cells are in a resting non-metastatic state at this time [58]. In a sustained hypoxic environment, tumor cells need to get more nutrients and oxygen to survive and proliferate. Then tumor cells increase cellular hypoxia inducible factor (HIF) transcription, thereby increasing blood vessel secrete growth factors (such as VEGF-A, VEGF-C) and chemokines (such as TNFα) to active endothelial cells. These growth factors stimulate blood vessels and lymphatic vessels to up-regulate the expression of specific integrins, such as αvβ3, α1β1, and α5β1, etc. Then these integrins have the ability to recruit some matrix metalloproteinases (MMPs, such as MMP2, MMP9) to degrade the basement membrane of vessels, thereby promoting endothelial cell migration and remodeling to form new blood vessels and lymphatic vessels. The new blood vessels not only provide nutrition to the tumor cells to continue to grow but also transfer the metabolic waste. Meanwhile, the blood vessels and lymphatic vessels provide a path for the local and distant metastasis of cancer cells [59]. However, only about 0.01% of cancer cells are capable of entering the circulation of metastasis to spread to distant sites, but this process is fatal for most cancer patients [57]. Initiation of metastasis begins with migration and invasion of cancer cells from the primary tumor into the surrounding tissues. To invade tissues, cancer cells undergo a pathophysiological transformation involving changes in the membrane characteristics, the process known as epithelial-mesenchymal transition (EMT) [60]. This process is followed by migration and invasion of cancer cells into the lymphatic system, and into the secondary tissue. To successfully produce a secondary tumor, cancer cells again transform back into the epithelial cell by mesenchymal-epithelial transition (MET). MET is required for anchoring of cancer cells to the surrounding tissues. Thus, the process of cancer cell metastasis is governed by many factors such as growth factors (basic fibroblast cell growth factor (bFGF), vascular epithelial growth factors (VEGF), membrane ion channels, cytokines, cell adhesion molecules, and extracellular matrix (ECM) [61]. In this section, we will mainly discuss two important membrane receptor proteins, i.e., membrane ion channels and cell adhesion molecule integrins that are widely studied as drug targets of metastasis or particularly EMT and MET of cancer cells.

#### Interaction with ion channels

In addition to changes in membrane electrostatic status, cancer cells also increase number of ion channels on the membrane especially voltage-gated channel such as sodium (Na^+^), potassium (K^+^), calcium (Ca^2+^), and chloride (Cl^-^) channels [62], and even the transient receptor potential (TRP) channels [63].These changes are required to compensate changes in membrane electrical charge and compensate the increase in proliferation rate and metabolic rate within the cancer cell. In normal cells, these voltage-gated channels maintain cellular homeostasis by controlling ion transport, volume regulation, and cell proliferation [62]. Over-expression of these channels is hypothesized to increase cell proliferation and render the cell tumorigenic. For example, potassium channel Kv11.1(hERG) expressed in heart, brain, etc. is known to be up-regulated in several cancers and its inhibition in cancer cells resulted in reduced proliferation and induction of apoptosis [62].

### Chloride channels

#### Chlorotoxin

Volumetric alteration of cancer cells is one of the features of invading cells [64]. The volumetric change observed in glioma cells is mainly regulated by ion and water channels such as chloride channels, aquaporin (AQPs). Malignant gliomas are a family of central nervous system (CNS) tumors, which are the most common type of primary brain tumor, and account for 80% of malignant tumors in the central nervous system (CNS) [65]. Glioblastoma (GBM) is one of the highest death rates of the brain tumor as the survival rate of its patients hardly exceeds sixteen months in the standard treatment [66]. Glioma cells express a high number of chloride channels that mediate EMT or MET, so as to achieve a suitable size and shape to cross the normal cells [65]. One of the significant development in last two decades is the discovery of chloride channel specific peptide-Chlorotoxin (CTX) from scorpion venom. This discovery has led to the development of glioma tumor- specific drug BLZ-100 known as “Tumor paint” [67].

CTX is a 4 kDa neurotoxin isolated from the venom of Israeli scorpion *Leiurus quinquestriatus* and can specifically recognize and block chloride channel [68, 69]. Thus, CTX inhibits EMT of glioma cells and prevent the invasion process. CTX is currently used to localize malignant glioma cells in the brain and also as a tool for delivery of therapeutic anti-tumor agents, for example, ^131^I [70], platinum [71], methotrexate [72], multifunctional nanoprobes +cDNA or siRNA [73], etc. CTX has 36 amino acids with three small antiparallel β-sheets packed against a α-helix and four disulfide bonds with the type C1–C4, C2–C6, C3–C7, and C5–C8 (Figure 2C) [68]. Its dense structure gives it the ability to cross the blood-brain barrier (BBB) and hence it is widely used against tumors on central nervous system. Through chemical modification of CTX and affinity chromatography other receptor targets such as matrix metalloprotease MMP-2 [74] and annexin A2 [75] have also been identified. In fact, the actual receptors of chlorotoxin need to be further study. Currently, CTX is mainly applied in analyzing brain glioma cancers.

**Figure 4:**
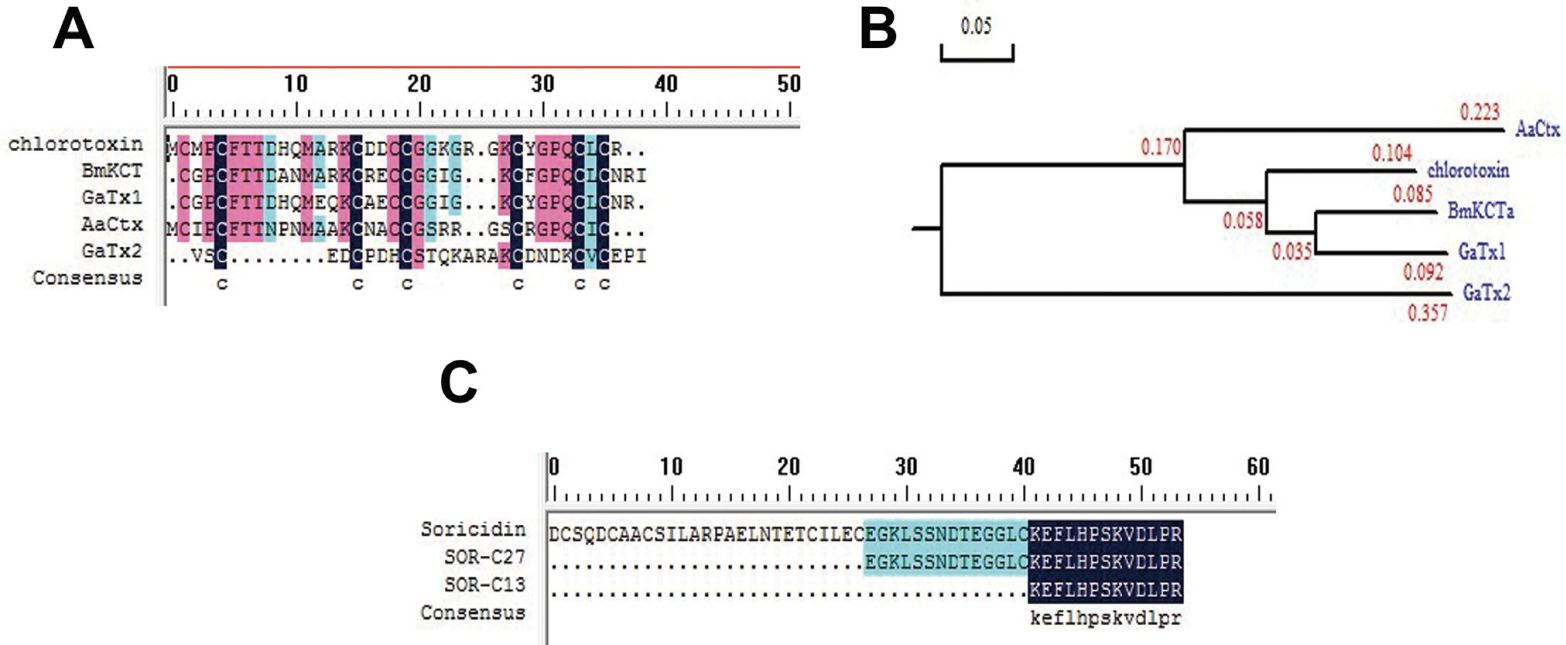
Chlorotoxin, Soricidin, and their related peptides (**A**) The sequence alignment of Chlorotoxin, BmKCTa, GaTx1, GaTx2, and AaCtx. (**B**) The phylogenetic tree of Chlorotoxin, BmKCTa, GaTx1, GaTx2, and AaCtx. From an evolutionary point of view, chlorine toxins, BmKCTa and GaTx1 may be relatively closer relationship than AaCtx and GaTx2. (**C**) The sequence alignment of Soricidin, SOR-C27, and SOR-C13.

^131^I-TM601 is iodinated on Tyr^29^ of CTX and has entered clinical trial phase I in 2002 in the USA [70]. The clinical trial is aimed to study its dosimetry of intracavitary-administered, safety, and biodistribution. It is an on-going trial because no major toxicity due to TM601 treatment is reported yet. The benefit of the treatment with TM601 would be a two-fold increase in the patient’s lifespan. ^131^I-TM601 CTX is currently in Phase III of the clinical trial [76, 77]. Further study in identifying peptides with similar properties has revealed some addition peptides in the venom of different species of scorpions. Today, many CTX -like peptides have been isolated, which includes peptide BmKCTa from the venom of *Buthus martenzii* scorpion [78, 79], peptides GaTx1 [80], and GaTx2 [81] both originating from the venom of *Leiurus quinquestriatus* scorpion, and peptide AaCtx from the venom of *Androctonus australis* scorpion [82] (Figure 4A). From an evolutionary point of view, chlorine toxins, BmKCTa and GaTx1 may be relatively closer relationship than AaCtx and GaTx2 (Figure 4B). The rBmKCTa showed a dose-dependent inhibitory role of the human glioma (SHG-44) cell growth (IC50 0.28 µM) with the blocking efficacy of rBmKCTa of 0.07–0.14 µM [83]. The dose is slightly lower than CTX (IC50 0.6 µM) [84]. AaCtx also inhibited glioma cell migration and tissue invasion in a dose-dependent manner with an IC50 value of 125 µM and 10 µM, respectively. Comparatively, BmKCTa has a greater possibility to become the next CTX in the near future.

#### Sodium and potassium channels

Analgesic-Antitumor Peptide (AGAP), also called antitumor peptide (ANTP), was purified from the venom of the Chinese scorpion *Buthus martensii* [85]. AGAP have 66 amino-acid residues with four disulfide bridges adopting the C1–C8, C2–C5, C3–C6, and C4–C7 pattern [86], which belongs to long-chain scorpion toxins recognizing sodium channels [87]. AGAP can anti-proliferation by arresting cell cycle at the G1 phase of human colon adenocarcinoma SW480 cells [88]. AGAP also inhibits the proliferation of gliomas cell SHG-44 [89].

According to the genes, sequence identity, and phylogenetic relationships of potassium channels, the family of voltage-gated potassium (K_v_) can be divided into K_v_1-K_v_12 by the International Union of Pharmacology (IUPHAR) [90]. Physiological functions of K_v_10.1 and K_v_11.1 are related to several cancers [90]. The hERG is a gene that codes for potassium channels K_v_11.1. Ergtoxin is a family of toxins composed of 42 to 62 amino acid residues from the venom of the Mexican scorpion, which can target K_v_11.1 channels [91, 92]. The proliferation of SKOV-3 ovarian cancer cell line was inhibited by ergtoxin (*p* < 0.05) and arrested cell cycle at the S and G2/M phase [93].

### TRP channels

#### Soricidin

Calcium signaling is another important regulator of cell proliferation. Altered calcium levels within the cells activate calcium sensitive pathways increasing tumor cell migration and metastasis [63]. Three transmembrane molecules (calcium channel ORAI1, stromal interaction molecule 1 (STIM1), and the transient receptor potential (TRP) channel family) that affect the balance of calcium ions have been identified in many tumors [94]. TRP vanilloid 5 and 6 (TRPV5 and TRPV6) belonging to TRP family has been implicated in tumor development and progression in many carcinomas of the ovary, prostate, thyroid, colon, and breast [94, 95]. Soricidin, a 54 amino acid toxin peptide isolated from the submaxillary salivary gland of the Shrew *Blarina brevicauda*, is reported to selectively inhibit TRPV6 channels [96, 97]. To reduce the paralytic activity and increase solubility and shelf stability, two additional peptides SOR-13 (13 amino acids) and SOR-C27 (27 amino acids) were synthesized from the C-terminus of soricidin (Figure 4C) [98]. SOR-C13 and SOR-C27 were shown to bind TRPV6 in ovarian cancer cells with high affinity [98]. In TRPV6 over-expressing cancer cells, both SOR-C13 and SOR-C27 are proved to inhibit calcium influx efficiently and reduce cell viability [98]. In 12 different cancer cell lines, both SOR-C13 and SOR-C27 decreased cell viability compared to chemotherapeutic drug cisplatin. SOR-C27 has been shown to be useful for treating human ovarian cancer in xenograft mouse *in vivo* [98]. In SKOV-3 ovarian cancer xenograft tumors in NUDE/SCID mice, SOR-C27 mice reduced the tumor volume significantly when compared to saline injected controls and was similar to carboplatin [98]. SOR-C27 and SOR-C13 are hypothesized to exhibit the anticancer activity through the activation of caspase 3 and caspase 7. Currently, SOR-C13 has completed phase 1 clinical trial in patients with over-expressing TRPV6 cancers [99].

### Interaction with cell adhesion molecules

#### Integrin and disintegrin family

Integrins are αβ heterodimeric transmembrane receptors that facilitate cell-extracellular matrix (ECM) adhesion, which is activated to mediate signaling pathways to affect cell survival, proliferation, control of transcription, and cytoskeletal organization [100]. Integrins are consist of 18 α subunits and 8 β subunits, so far known to assemble into 24 distinct integrins. According to receptor specificity, integrins can be divided into 4 subfamilies, including RGD receptors, collagen receptors, laminin receptors, and leukocyte-specific receptors. Currently, RGD receptors gain more research attention because they are related to several cancers, thrombosis coagulation and other diseases [101], which include 8 integrins (α_V_β_1_, α_V_β_3_, α_V_β_5_, α_V_β_6_, α_V_β_8_, α_IIb_β_3_, α_5_β_1_, and α_3_β_1_). Integrin family is observed to play a pivotal role in cancer cell metastasis and as well as the survival of cancer cells outside the tumor environment [102, 103], in which α_V_β_3_ plays a vital role in angiogenesis. The integrin α_V_β_3_ was observed to up-regulate expression in the following cancer progression, such as melanoma, glioblastoma, breast cancer, prostate cancer, pancreatic cancer and ovarian cancer [102]. So the α_V_β_3_ is used as a targeted molecule for cancer drugs, for example, cilengitide [104] Hence, integrin family is one of widely studied drug targets in cancer therapy [102]. Integrin antagonists “disintegrin” from snake venom is currently being explored and also has been proven in experimental studies to be a good candidate for anticancer drug development [105].

Disintegrin is the name given to a family of non-enzymatic, small molecular weight cysteine-rich proteins found in the venoms of few snake species [106]. Based on the length and the number of disulfide bonds, the disintegrin family can be divided into five different classes, i.e., Short, Dimeric, Medium-size, Long, and Cysteine-rich disintegrins [107]. It can also be classified based on their interacting tripeptide motifs such as RGD (also include VGD/MGD/WGD/KGD), MLD, ECD and KTS (RTS) which determines its selectivity to integrins [108, 109]. Snake venom disintegrins interact with integrins and block their functional ability to inhibit cancer cells metastasis. Three decades ago, Arg-Gly-Asp-Ser (RGDS) was found to be as the minimal binding site in fibronectin [110], and later it has been found that serine substitution with other amino acids does not affect its biological function [111]. And then later it was found that the RGD motif not only exists in the fibronectin but also in other extracellular matrixes (such as vitronectin, fibrinogen, osteopontin, etc.) and snake venom disintegrins as an active site [112, 113]. These studies have led to the development of many RGD-based strategies in cancer drugs therapies and diagnoses such as RGD antagonists, RGD conjugates and RGD nanoparticles [58]. In the last decade, many disintegrins have been shown to inhibit cancer cell migration and angiogenesis during metastasis of cancer cells. Table 3 shows some of the examples of disintegrins and disintegrin-like proteins which are shown to be an anti-angiogenic effect, including Salmosin [114], Albolabrin [115], Alternagin-C [116], Obtustatin [117], Contortrostatin [118, 119], Lebein [120], Jerdostatin [121], Rhodostomin [122], Saxatilin [123], Triflavin [124], Acurhagin-C [125], and Lebestatin [126]. For example, Salmosin, about 8.0 kDa peptide of novel disintegrin, is isolated from the venom of snake *Agkistrodon halys brevicaudus* [127, 128].

**Table 3:** List of disintegrins and disintegrin-like proteins with anti-angiogenic effect

Disintegrin	Snake species	Molecular weight	Functional class	References
Salmosin	*Agkistrodon halys brevicaudus*	8.0 kDa	RGD	[114]
Albolabrin	*Trimeresurus albolabris*	7.5 kDa	RGD	[115]
Alternagin-C	*Borthops alternatus*	29.0 kDa	ECD	[116]
Obtustatin	*Viperalebetina obtuse*	4.4 kDa	KTS	[117]
Contortrostatin	*Agkistrodon contortrix*	13.5 kDa	RGD	[118, 119]
Lebein	*Macrovipera lebetina*	7.0 kDa	RGD	[120]
Jerdostatin	*Protobothrops jerdonii*	4.0 kDa	RTS	[121]
Rhodostomin	*Calloselasma rhodostoma*	7.0 kDa	RGD	[122]
Saxatilin	*Gloydius halys*	7.7 kDa	RGD	[123]
Triflavin	*Protobothrops flavoviridis*	7.57 kDa	RGD	[124]
Acurhagin-C	*Agkistrodon acutus*	1.3 kDa	ECD	[125]
Lebestatin	*Macrovipera lebetina*	4.4 kDa	KTS	[126]

**Figure 5:**
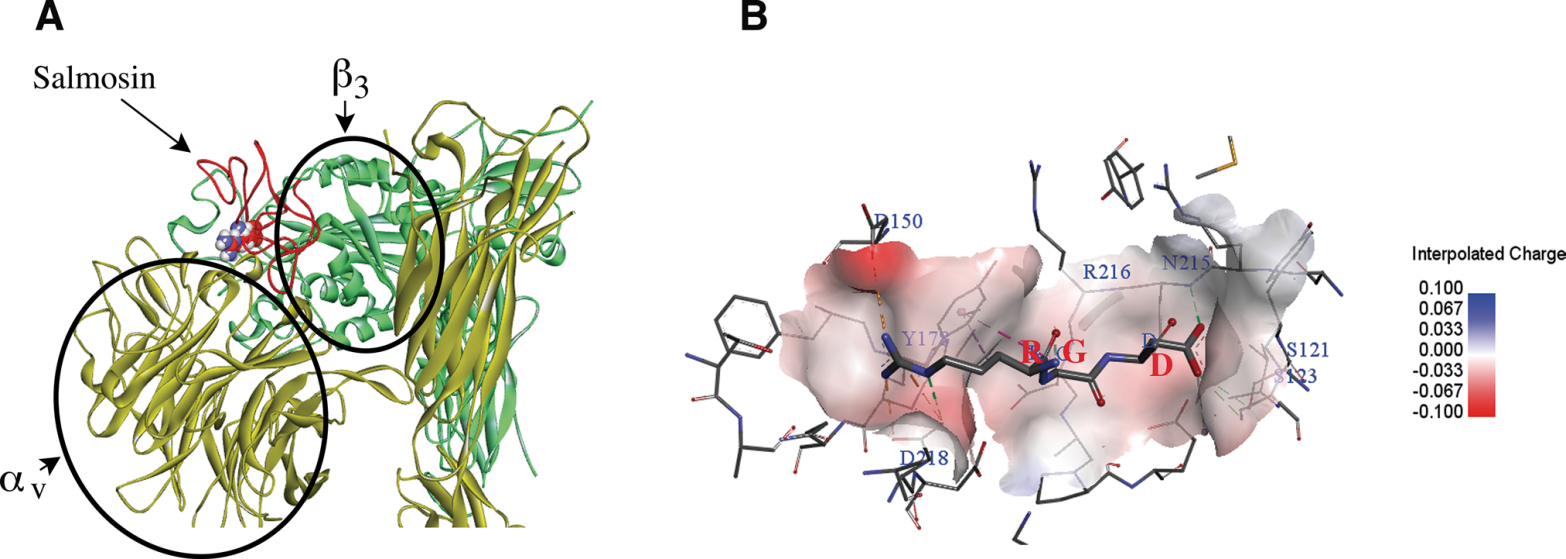
Molecular modeling between Integrin α_v_β_3_ (PDB: 1JV2) and disintegrin salmosin (PDB: 1L3X) based on the crystal structure of α_v_β_3_ complex (PDB: 1L5G) (**A**) Ribbon modeling is depicting the interaction between salmosin and integrin α_v_β_3_ receptor. Salmosin binds to the hinge created between α_v_ and β_3_ subunits of integrin. Yellow-subunit α_v_, green-subunit β_3_ and red-salmosin (the ball structure represents the RGD site). (**B**) The integrin α_v_β_3_ receptor surface of the RGB motif interactions are shown in the charge mode (PDB 1L5G). Figures were constructed by Discovery Studio version 2016.

Salmosin was purified from Korean snake, which has 73 amino acids polypeptide with a proposed adhesive protein integrins recognition site Arg-Gly-Asp (RGD) [128]. Salmosin, an antagonist of glycoprotein (GP) IIb-IIIa, acts as a platelet aggregation inhibition factor and is known to inhibit capillary endothelial cell proliferation and angiogenesis [128]. Salmosin inhibits the proliferation of metastatic cancer cell by specifically binding to α_v_β_3_ integrin on bovine capillary endothelial cells. Salmosin also inhibits angiogenesis function by specifically down-regulating basic fibroblast growth factor (bFGF)-induced bovine capillary endothelial cell proliferation [114, 127]. We used the software of Discovery Studio 2016 to construct the interaction picture of molecular modeling between Salmosin (PDB: 1L3X) [127] and α_v_β_3_ (PDB: 1JV2) [129] (Figure 5A) based on the crystal structure of α_v_β_3_ complex (PDB: 1L5G, Figure 5B). In order to highlight the binding site RGD between salmosin and integrin αvβ3, Figure 5 shows only the partial structure of αvβ3. Salmosin identified to inhibit glycoprotein (GP) IIb-IIIa binding to immobilized fibrinogen with an IC50 of 2.2 nM and ADP-induced platelet aggregation with an IC50 of 131 nM [128].

Vicrostatin (VCN) is a synthetic disintegrin that contains 69 amino acids consisting of the 6 amino acids of the C-terminal tail of disintegrin echistatin and 63 amino acids of N-terminal of recombinant disintegrin contortrostatin [130]. VCN can bind to several integrin ligands, such as α_v_β_3_, α_v_β_5_, and α_5_β_1_. A formulation of VCN can significantly reduce the growth and dissemination ovarian cancer cells [131]. VCN also inhibits the proliferation of gliomas cells [132]. For the last two decades, peptidomimetic has been widely applied to drug design, for example, Cilengitide [104], ATN-161 [133], and GLPG0187 [134].

#### Interaction with gastrin-releasing peptide receptor

The gastrin-releasing peptide receptor (GRPR) belongs to G protein-coupled receptor. Its endogenous ligand is gastrin releasing peptide [135]. Bombesin is a 14-amino acid peptide isolated from the skin of the toad *Bombina bombina* [136] and is a homolog of the gastrin-releasing peptide. In clinical trials, bombesin was labeled by 68Ga of gallium as the BAY86-7548 drug to evaluate primary and metastatic prostate cancer [137]. The sensitivity for detection of primary and metastatic prostate cancer is 88% and 70%, respectively. Currently, the BAY86-7548 has entered phase II/III (the information is from https://clinicaltrials.gov/ct2/show/NCT02624518?term=BAY86-7548&rank=2).

#### Interaction with other membrane receptors

Most types of cancers acquire metastatic function by enhancing growth signals, tissue invasion, and angiogenesis. This process requires the regulation of metalloproteinase (the metal involved in the catalytic reaction of the enzyme belongs to the metalloproteinase). Metalloproteinase has exopeptidases and endopeptidases subgroups, in which endopeptidase includes ADAM (a disintegrin and metalloproteinase) proteins and matrix metalloproteinases [138]. Several ADAM can active special membrane receptor-related cancers, such as ADAM10 can active HER2 receptor (target molecule of breast cancer) [139]. Jararhagin (52.0 kDa) [140] and Jararhagin C (a derivative of Jararhagin, 28.0 kDa) [141] from *Bothrops Jararaca* are few examples of venom proteolytic enzymes. Their mechanism of action involves proteolytic cleavage of growth factors and corresponding receptor, thereby degradation of extracellular matrix and inhibiting growth signaling.

#### Metalloprotease-Jararhagin

Jararhagin, a 52.0 kDa snake venom metalloprotease, is isolated from snake *Bothrops Jararaca* and its 3D structure is modeled from SWISS-MODEL (Figure 2E) [140, 142]. It is a multidomain toxin with a catalytic Zn-dependent metalloproteinase domain, ECD/disintegrin-like domain and a cysteine-rich domain [143]. Jararhagin is known to stimulate cytoskeletal rearrangement in normal epithelial cells. In human melanoma cells Sk-Mel-28, *in vitro* treatment of jararhagin decreased tumor cell invasion along with inhibiting proliferation and reducing cell viability [144]. Upon addition of jararhagin tumor cells acquired stress-induced morphological characteristics such as round shapes, cluster formation in suspension and had decreased cell viability [144]. Inhibition of catalytic Zn-dependent metalloproteinase domain (further referred as Jari- a catalytic domain inactivated with 1,10-phenanthroline) reduced cell adhesion and viability of tumor cells compared to its native form [145]. In B16F10 murine melanoma cells, jari induced cell detachment, apoptosis, and necrosis which was confirmed by SEM imaging [145]. Inhibition of catalytic domain (Jari) increased the anti-tumor effects suggesting a possible role of ECD/disintegrin-like domain or cysteine-rich domains. Additionally, jararhagin induced caspase 3 activation, apoptotic body formation, DNA fragmentation, and chromatin condensation in B16F10 cells [145]. The similar apoptotic effect was observed in SK-Mel-28 human cells, murine endothelial cells, and HUVEC cells treated with jararhagin. Jararhagin also reduced tumor metastasis and induced G0/G1, G2/M cell cycle arrest in lung carcinoma, B16F10 cells, and Sk-Mel-28 cells.

### Interactions with cell signaling cascade

Although cellular signaling process involves the complex network of protein interactions, genetic mapping of various types of tumors has shown up-regulation of either MAPK signaling pathway (ERK1/2, JNK, p38) or PI3K/AKT pathway that decides cells fate and cancer progression. In many cancer cell lines, anti-tumor agents are known to alter the activities of MAPK signaling pathway [146, 147]. Activator or stimuli for initiating these pathways can relay from either external mitogen through transmembrane receptor tyrosine kinase (RTK) (promotes cell survival/anti-apoptotic) or by internal genetic aberration stress (pro-apoptotic). Uncontrolled activation of these pathways in response to external stimuli is observed in many cancer types. Upon RTK stimulation by external mitogens, a cytosolic protein Ras gets phosphorylated and initiates either MAPK signaling pathway or PI3K/AKT pathway. Extracellular signal-regulated kinase (ERK) activation controls many cellular processes including gene expression, cell proliferation, etc. however, ERK 1/2 up-regulation is known to induce apoptosis. In contrast, phosphatidylinositide 3-kinase (PI3K) pathway phosphorylates inositol lipids on the plasma membrane in 3-position converting phosphatidylinositol-4,5-bisphosphate to phosphatidylinositol-3,4,5-trisphosphate which allows attachment and activation of many other signaling proteins. Akt and mTOR are the downstream target molecules of the PI3K pathway which regulates cell cycle and cell viability by anti-apoptotic gene expression. The exact mechanism of ERK/MAPK in apoptosis in not well understood yet. It is hypothesized that the DNA-damaging stimuli induce ERK-mediated expression of pro-apoptotic factors in neuronal cells. Some of the venom peptides are known to promote ERK/MAPK and inhibit PI3K/AKT pathway thereby causing cell death.

#### MAPK signaling pathways

Bengalin, a 72.0 kDa peptide isolated from the venom of Indian black scorpion *Heterometrus bengalensis* Koch [148], is known to have a particular anti-proliferative effect in human leukemic U937 cells [149]. Treatment of bengalin to U937 cells induced upregulation of ERK 1/2 expression and downregulation of AKT thereby inducing apoptosis. Bengalin induced caspase-3 activation is observed to induce apoptosis. Bengalin treatment was specific to MAPK ERK 1/2 and did not alter JNK or p38 [149]. Further, inhibiting ERK 1/2 and caspase 3 using blockers before bengalin treatment lead to activation of autophagy pathway in U937 cells. Bengalin increased autophagy markers such as Beclin-1, Atg12, Atg7, Atg5, and Atg3 in U937 cells [149]. Another peptide NVP(1), a 6.6 kDa of molecular weight isolated from the venom of Wasp *Nidus vespae* [150], is also known to interact with MAPK signaling cascade. NVP(1) induced cell cycle arrest in HepG2 hepatoma cells and inhibited the mRNA expression of cyclin B, cyclin D1, and cyclin E. NVP (1) was observed to cause nuclear chromatin condensation of HepG2 cells and activation of ERK1/2 to induce apoptosis.

#### PI3K/Akt signaling pathway

The PI3K/Akt pathway represents a central survival-related signal transduction pathway, and its activation enhances cell survival and promotes tumor invasion [151]. However, an increased expression of PI3K/AKT pathway is shown to induce lymphatic cancer in mice. Similarly, activated PI3K/AKT pathway was also observed in the variety of human cancer types [146]. Inhibition of PI3K in Hodgkin lymphoma (HL)-derived cell lines using PI3K inhibitors induced G0/G1 cell cycle arrest and activated caspase-3 induced apoptosis [152]. Similar effects were observed when peptides extracted from scorpion *Buthus martensii Karsch* (BmK) venom was treated against human lymphoma Raji and Jurkat cells and breast cancer MCF-7 cells [153, 154]. Peptides from BmK venom-induced caspase-3 up-regulation, Bcl-2 down-regulation and decreased cell cycle related protein cyclin D in MCF-7 cells and human lymphoma cells [153]. To elucidate the possible mechanism, Gao et al. used two different human lymphoma cell lines Raji and Jurkat cells that were either PTEN positive or PTEN negative [154]. PTEN (phosphate and tensin homolog) is a critical tumor suppressor gene known to inactivate Akt kinase. Peptides from BmK venom increased expression of PTEN mRNA only in Raji cells which negatively regulated Akt and induced apoptosis in Raji cells [154]. In continuation peptides from BmK venom also induced cell cycle arrest in both cell lines by increasing expression of p27 (a cyclin- dependent kinase 1 protein).

#### Other mechanism

Apoptosis is a form of the programmed cell death, which plays a major role in cellular activities [155]. A family of cytosolic protease known as caspases is the primary inducers of apoptosis within the cell. Caspases such caspase 3, caspase 9, and caspase 12 are activated in the presence of cellular stress leading to apoptosis. Cellular stress executed through disruption of cell organelle endoplasmic reticulum and mitochondria induces the release of calcium, glucose, and cytochrome c, which activates caspases [156]. Cellular stress due to DNA fragmentation also activates caspases. Cardiotoxin III (CTX III) with 60 amino acids (Figure 2D) isolated from snake *Naja naja atra* venom is observed to induce an anti-cancer effect on human colorectal cancer Colo205 cells through mitochondrial apoptotic pathway [157, 158]. In human leukemia (HL-60 cells) CTX III is also observed to induce endoplasmic reticulum stress thereby releasing calcium and glucose-related protein 78 (GRP78), which induces caspase 12 induced apoptosis [159]. Besides, CTX III also initiated the mitochondrial apoptotic pathway evidenced by an increased Bax/Bcl-2 ratio, the release of cytochrome c, and activation of caspase 9 [158]. In breast cancer MCF-7 cells, CTX III was also observed to up-regulate pro-apoptotic marker Bax, release cytochrome c and inactivate nuclear factor-kappa B (NF-kB) leading to suppression of proliferation and induction of apoptosis [160]. Thus CTX III induces apoptosis by both endoplasmic reticulum stress and a mitochondrial stress.

## VENOM PEPTIDES IN IMMUNE MODULATION

Immune reaction represents the primary defense system against non-self components including cancer cells in an organism. In brief, the initial response to non-self components is mediated by innate immune response cells such as natural killer cells (NK) and antigen presenting cells (APC) followed by adaptive immune response mediated by T cells and B cells. Snake, bee, and wasp venoms are known to enhance both innate and adaptive immune response in parallel to inducing a cytotoxic effect within the cells [161]. In some studies, bee venom is known to the by-pass innate immune system and directly influences T cell activity or adaptive immune system [161, 162]. Venom peptides such as cobra venom factor, cobra toxin, PLA2, melittin from snake *Naja naja atra* were observed to enhance NK cells activity by increasing the production of cell stress signaling protein interferon-g in immune-suppressed mice (IFN-g) [163]. In the same study, authors have also observed suppression of T cell proliferation especially CD8 T cells through inhibition of NF-κB (nuclear factor kappa-light-chain-enhancer of activated B cells) [163]. In contrast, bee venom PLA2 and melittin are reported to increase T cell response by increasing synthesis of cytokines IL-1 and TNF-a on monocytes [164].

In cancer therapy, immune reactions by the complementary system have also been observed to mediate clearance of dead cells and influence inflammation [165, 166]. The complementary system enhances initial recognition of immune cells to cancer cells and its considered to be a part of immunosurveillance response against cancer [165]. The complementary system is considered to complement innate immune response either by complement-dependent cytotoxicity or through antibody-dependent cell mediated cytotoxicity. However, complementary cascade protein C5 activation is observed to inhibit CD8+ T cell [165]. Furthermore, mice deficient of protein C3 or C4 showed a decrease in the proliferation rate of lymphatic TC-1 cell line [165]. These studies have linked the loss of complementary system to suppress the immune response. Moreover, the complementary system is observed to prevent apoptosis, increase growth factor signaling, and enhance angiogenesis. Snake venom protein such as cobra venom factors and humanized cobra venom factor (hCVF) inhibit complement system [167–169]. Complement system inhibition has also increased expression of chemokine CCL5, CXCL10, and CXCL11. Although the role of the complement system is still being studied, its importance in various type of cancer and its interaction with immune cells is yet to be analyzed.

## FUTURE PROSPECTS

Peptide based targeted therapy has gained momentum in the last two decades. Smaller size and tumor penetrating ability makes peptides an ideal choice for targeting cancer cells. Among many, peptide based anticancer drugs in market three peptides Leuprolide, Octreotide and Goserelin have reached a global sale of 1 billion US dollar per year [170]. Today, a wide range of synthetic peptides have been tested for their anticancer abilities among which some are in clinical trials. Synthetic peptides such as Cilengitide, IM862, ATN 161, and angiotensin-(1–7), etc. are being tested against various types of cancers. Synthetic peptides with cancer cell specificity can also be used to deliver a cytotoxic drug, or as hormone antagonist, or even as a vaccine in reducing or stimulating immune reactions within the system. Recent studies on molecular aspects of cancer development and progression have also offered prospects in both identifying potential targets and designing potential ligands [109]. Many of potential anti-cancer peptides such as stimuvax, primovax, melanotan are in clinical trials for elucidating efficiency, bioavailability, and metabolism [8]. Therefore, Venom based peptides have a wide range of application in modern biology from diagnostic to the treatment of the disease [10]. For example, Chlorotoxin analog drug BLZ-100 tagged with a fluorescence dye is able to lights up only cancer cells in brain tumor so that it can be precisely excised from the brain [11]. BLZ-100 is also known as “Tumor paint” is currently in phase 1b clinical study.

Furthermore, advancement in proteomics and genomics approach has made possible to isolate and characterize the potential anticancer peptides from venom pool. High throughput screening using mass spectrometry is very useful to venom characterization as it can read low concentration peptides along with generating mass datasets that could be analyzed further. Similar sequencing RNA isolates from venom glands will provide generate the pool of expressed proteins and peptide database. Currently, many research labs are combining mass spectrometry mass datasets with next generation RNA sequencing to analyze venom and identify novel biologically active peptides [171]. In continuation, parallel to this advancement, today it is possible to tailor a peptide to mimic or antagonize biological activity of a natural peptide, i.e., by advanced peptidomimetic technique. Peptidomimetic approach promotes future use of peptide-based drugs by (i) increasing the stability of peptides against chemical degradation and enzymatic degradations leading to increasing lifetime within the biological system; (ii) decreasing the size of peptides making the molecule smaller and easily accessible for interaction; and (iii) by managing the electrostatic charge distribution and polarity of peptide that is important for peptide interactions. These developments, i.e., large scale production and tailoring of peptides to make cancer treatment affordable to patients, has created a “new wave” in design and discovery of anti-cancer drugs.

Nanotechnology has brought entirely new perspectives in the preparations of peptide-based drugs, for example, venom extracts from snake *Walterinnesia aegyptia* tagged with silica nanoparticle enhanced the proliferation of immune cell as well as decreased the proliferation of human breast cancer cell [172]. There are other examples, snake toxin neurotoxin-loaded polylactic acid nanoparticles [173] and CTX-conjugated iron oxide nanoparticles [174]. These polymer nanoparticles and iron superoxide nanoparticles have enhanced the prospects of using the venom peptides.

Exploring venom peptides tends to be more beneficial in targeted therapy. Venom peptides are smaller, specific and in some cases can penetrate cell membrane inducing the cytotoxic effect. Some of the hurdles of antibodies such as bioavailability, cross reactivity, degradation can be eliminated using synthetic peptides. Currently, venom peptides have also found useful as “Guiding” ligands for nano-vectors towards cancer cells. Many of nano-vectors were used for gene therapy and therapy against cancer by taking chlorotoxin as targeting ligand. Chlorotoxin nano-vectors carrying methotrexate agent has shown an increased cytotoxicity against glioma cells along with an increase in contrast imaging of tumors in mice [174]. Chlorotoxin guided the delivery of nano-vectors carrying enhanced green fluorescence protein (EGFP) has shown an increased gene delivery and expression of EGFP in C6 glioma cell line [175, 176]. In addition, expression of venom peptides such as Salmosin, melittin, phospholipase A2 in cancer cells by gene therapy has also been analyzed [177, 178]. Chlorotoxin loaded targeted nano particles carrying melittin gene has shown higher gene transfection efficiency along with decreased cell viability in pancreatic cancer cells [178]. This advance nanotechnology has brought entirely new perspectives in the preparations of peptide-based drugs and enhanced the prospects of using the venom peptides.

## CONCLUSIONS

Initial interaction of venom peptides with the target molecule is the first and foremost step which plays a key role in venom-peptide induce anti-cancer activity. Many of the examples used in this review emphasize the importance of this initial step. This interaction is guided either by the polarity of a molecule or by specific pharmacophore domain. Followed by initial interactions, peptides tend to exhibit their effects mostly by membrane interactions, although many other mechanisms such as intracellular peptide-protein interaction, peptide-DNA interactions are still existing. The targeted therapy drug with the specificity on certain molecule determines the limitation of its use, for example trastuzumab can only be used for HER2-positive breast cancer which occupy about 20% of breast cancer patients. Drugs derived from venom are no exception. Currently, venom-based drugs such as chlorotoxin and integrin αvβ3 drugs are used mainly in brain tumor and cancer with overexpressed αvβ3, respectively. Understanding mechanism of action of venom-peptides helps to curate a “staple peptide” with increased specificity in various types of cancer cells. As the molecular interaction of each venom peptide may vary, each peptide needs to be evaluated for its therapeutic potential. This review on venom peptides in cancer therapy fortifies our current understanding of their molecular mechanism of action and paves the way for better utilization of venom-based drugs.

## Abbreviations

FDA: Food and Drug Administration
ACE: Angiotensin-converting enzyme
PS: phosphatidylserine
PE: phosphatidylethanolamine
AMPs: antimicrobial peptides
Cyt C: cytochrome C
EndoG: endonuclease G
SMAC: second mitochondria-derived activator of caspases
TP53/P53: Tumor protein p53
CDKN1A: cyclin-dependent kinase inhibitor 1
GADD45α: Growth arrest and DNA-damage-inducible 45 alpha
MDM2: Mouse double minute 2 homolog
Bcl-2: B-cell lymphoma 2
CAS-3: CRISPR associated protein 3
CAS-7: CRISPR associated protein 7
PLA2s: Phospholipases A2
HUVECs: human umbilical vein endothelial cells
HPAECs: human pulmonary artery endothelial cells
VEGF: Vascular endothelial growth factor
bFGF/FGF2: Basic fibroblast growth factor
CAM: chorioallantoic membrane
HIF: hypoxia inducible factor
MMPs: matrix metalloproteinases
EMT: epithelial-mesenchymal transition
MET: mesenchymal-epithelial transition
ECM: extracellular matrix
TRP: transient receptor potential
CNS: central nervous system
GBM: Glioblastoma
AGAP: Analgesic-Antitumor Peptide
IUPHAR: International Union of Pharmacology
GRPR: Gastrin-releasing peptide receptor
ADAM: a disintegrin and metalloproteinase
HER2: human epidermal growth factor receptor 2
MAPK: mitogen-activated protein kinase
ERK: extracellular signal–regulated kinases
PI3K: Phosphatidylinositol-4,5-bisphosphate 3-kinase
Akt/PKB: Protein kinase B
PTEN: Phosphatase and tensin homolog
CTX III: Cardiotoxin III
GRP78: glucose-related protein 78
NF-kB: nuclear factor-kappa B
NK: natural killer cells
APC: antigen presenting cells
CD8: cluster of differentiation 8
GFP: Green fluorescence protein
